# The genome sequence of the Common Marble, *Celypha lacunana* (Denis & Schiffermüller, 1775) (Lepidoptera: Tortricidae)

**DOI:** 10.12688/wellcomeopenres.24736.1

**Published:** 2025-08-11

**Authors:** Adrian Gardiner, Gavin R. Broad, Laura Sivess, Stephanie Holt, Natural History Museum Genome Acquisition Lab, Darwin Tree of Life Barcoding collective, Wellcome Sanger Institute Tree of Life Management, Samples and Laboratory team, Wellcome Sanger Institute Scientific Operations – Sequencing Operations, Wellcome Sanger Institute Tree of Life Core Informatics team, Tree of Life Core Informatics collective, Darwin Tree of Life Consortium

**Affiliations:** 1 Natural England, Norwich, England, UK; 2 Natural History Museum, London, England, UK

**Keywords:** Celypha lacunana, Common Marble, genome sequence, chromosomal, Lepidoptera

## Abstract

We present a genome assembly from a male specimen of *Celypha lacunana* (Common Marble; Arthropoda; Insecta; Lepidoptera; Tortricidae). The genome sequence has a total length of 591.83 megabases. Most of the assembly (99.68%) is scaffolded into 27 chromosomal pseudomolecules. The mitochondrial genome has also been assembled, with a length of 17.21 kilobases.

## Species taxonomy

Eukaryota; Opisthokonta; Metazoa; Eumetazoa; Bilateria; Protostomia; Ecdysozoa; Panarthropoda; Arthropoda; Mandibulata; Pancrustacea; Hexapoda; Insecta; Dicondylia; Pterygota; Neoptera; Endopterygota; Amphiesmenoptera; Lepidoptera; Glossata; Neolepidoptera; Heteroneura; Ditrysia; Apoditrysia; Tortricoidea; Tortricidae; Olethreutinae; Olethreutini; *Celypha*; *Celypha lacunana* (Denis & Schiffermüller, 1775) (NCBI:txid1594247)

## Background

*Celypha lacunana* ([Denis & Schiffermüller], 1775) is a tortricid moth that has recently been given the English common name of Common Marble ( Sterling *et al.*, 2023; Wheeler, 2017). The genus name is derived from the Greek *celyph* meaning husk, rind or shell ( Bug Guide, 2025), with the species name derived from the Latin *lacuna* meaning gap or cleft, which refers to a notched marking in the median fascia ( Norfolk Moths, 2025).

The adult moths have a wingspan of 14–18 mm and are very variable in colouration. The forewings are largely dark brown flecked with paler yellowish or greyish brown marks. They have two creamy or silvery cross bands from the costa, one at a third and one just after one half. The notch (or lacuna) occurs on the outer edge of the inner cross band, extending into the middle of the wing, but rarely reaching the second cross band ( British Lepidoptera, 2025; Sterling *et al.*, 2023). A more uniform variation occurs with the forewing blackish, speckled grey. The hindwing is greyish brown with a narrow off-white sub-terminal band and a darker grey-brown terminal band. Once familiar with the species, it is difficult to confuse with other species if the specimen is reasonably fresh. The most similar species in the British and Irish Isles is *Celypha rurestrana*, but this is a rare species of the south-west and south Wales, where the cross bands are whiter, the outer edge of the inner cross brand straighter and the notch much less distinct ( Sterling *et al.*, 2023). The male and female genitalia of *C. lacunana* are both distinctive and unlikely to be confused with any other species ( Moth Dissection, 2025).

The larvae hatch from August and overwinter as an early instar, with feeding starting again in the spring. They feed within folded and spun leaves fastened with silk and use a very wide range of herbaceous plants ( Smart, 2021).

*Celypha lacunana* is predominantly a species of the Western Palearctic ( GBIF Secretariat, 2025), with records as far east as western Russia and the eastern end of the Black Sea. There are a few recent records from eastern and western Canada, which are presumed to be native rather than introduced ( Gilligan *et al.*, 2020). It is one of the most common tortrix moths in the British and Irish Isles, recorded from every vice county in Britain and Ireland, including the Western Isles, Shetland and Orkney ( Hancock, 2014).

*Celypha lacunana* occurs in a wide range of habitats and is regularly found in gardens, hence its wide distribution. Adult moths are mostly on the wing between May and August in Britain, where they can be disturbed during the day, but are most active at dusk and through the night when they are often attracted to light ( Sterling *et al.*, 2023; UK Moths website, 2025).

**Figure 1.**
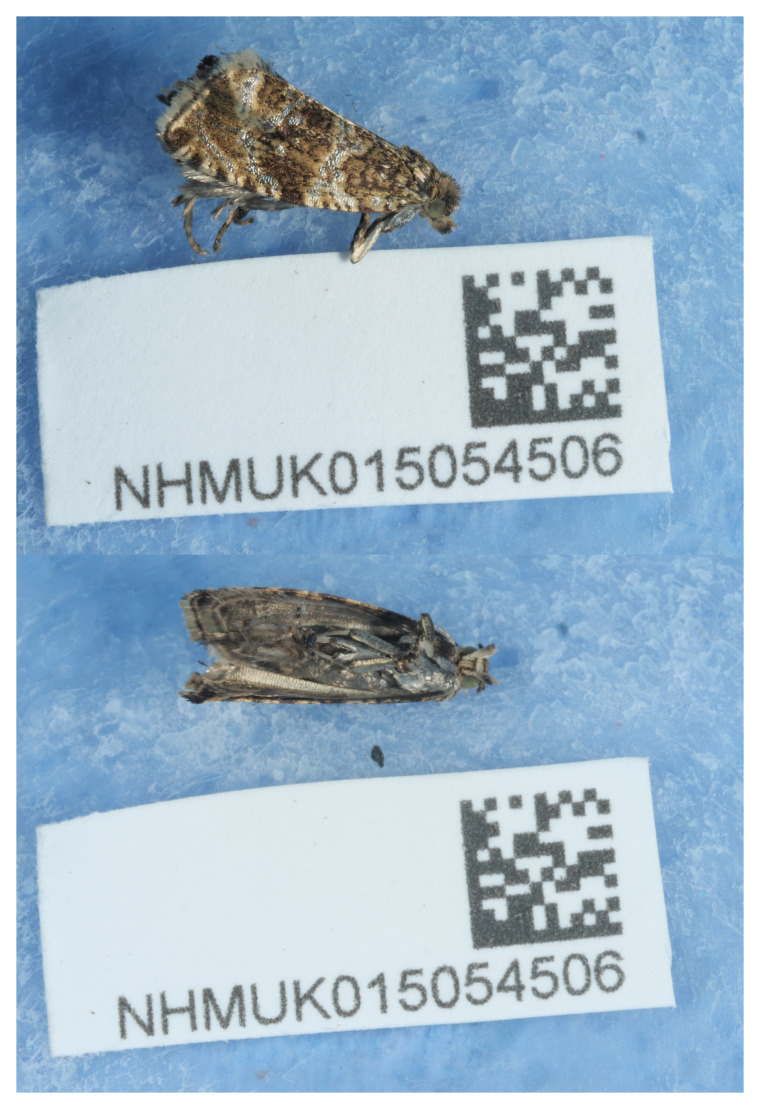
Photograph of the Celypha lacunana (ilCelLacu3) specimen from which samples were taken for genome sequencing.

The specimen used for this sequence ( Figure 1) was caught in an actinic moth trap at Winterton Dunes National Nature Reserve on the night of 28 June 2022 The trap was located on the edge of a sallow block in semi-fixed dunes dune heath. This trapping took place during the annual Natural England Natural History Museum BioBlitz to add species to the Darwin Tree of Life and UK Barcode of Life projects. Natural England’s involvement in DNA research is focussed on species detection, increased understanding of species assemblages for nature conservation, and to link with other environmental data to facilitate greater understanding of ecosystems and how they function. High quality reference sequences are essential to this work.

## Methods

### Sample acquisition and DNA barcoding

The specimen used for genome sequencing was an adult male *Celypha lacunana* (specimen ID NHMUK015054506, ToLID ilCelLacu3; Figure 1), collected from Winterton Dunes, England, United Kingdom (latitude 52.73, longitude 1.69) on 2022-06-28. The specimen was collected and identified by Adrian Gardiner (Natural England). A second specimen was used for Hi-C sequencing (specimen ID NHMUK014536856, ToLID ilCelLacu2). It was collected from Gilbert White’s House, Selborne, England, United Kingdom (latitude 51.09, longitude –0.94) on 2021-06-10. The specimen was collected by Gavin Broad, Laura Sivess and Steph Holt and identified by Gavin Broad (Natural History Museum). Sample metadata were collected in line with the Darwin Tree of Life project standards described by Lawniczak *et al*. (2022).

The initial identification was verified by an additional DNA barcoding process according to the framework developed by Twyford *et al.* (2024). A small sample was dissected from the specimen and stored in ethanol, while the remaining parts were shipped on dry ice to the Wellcome Sanger Institute (WSI) (see the protocol). The tissue was lysed, the COI marker region was amplified by PCR, and amplicons were sequenced and compared to the BOLD database, confirming the species identification ( Crowley *et al.*, 2023). Following whole genome sequence generation, the relevant DNA barcode region was also used alongside the initial barcoding data for sample tracking at the WSI ( Twyford *et al.*, 2024). The standard operating procedures for Darwin Tree of Life barcoding are available on protocols.io.

### Nucleic acid extraction

Protocols for high molecular weight (HMW) DNA extraction developed at the Wellcome Sanger Institute (WSI) Tree of Life Core Laboratory are available on protocols.io ( Howard *et al.*, 2025). The ilCelLacu3 sample was weighed and triaged to determine the appropriate extraction protocol. Tissue from the whole organism was homogenised by powermashing using a PowerMasher II tissue disruptor. HMW DNA was extracted using the Automated MagAttract v2 protocol. DNA was sheared into an average fragment size of 12–20 kb following the Megaruptor®3 for LI PacBio protocol. Sheared DNA was purified by automated SPRI (solid-phase reversible immobilisation). The concentration of the sheared and purified DNA was assessed using a Nanodrop spectrophotometer and Qubit Fluorometer using the Qubit dsDNA High Sensitivity Assay kit. Fragment size distribution was evaluated by running the sample on the FemtoPulse system. For this sample, the final post-shearing DNA had a Qubit concentration of 14.8 ng/μL and a yield of 1 924.00 ng.

### PacBio HiFi library preparation and sequencing

Library preparation and sequencing were performed at the WSI Scientific Operations core. Libraries were prepared using the SMRTbell Prep Kit 3.0 (Pacific Biosciences, California, USA), following the manufacturer’s instructions. The kit includes reagents for end repair/A-tailing, adapter ligation, post-ligation SMRTbell bead clean-up, and nuclease treatment. Size selection and clean-up were performed using diluted AMPure PB beads (Pacific Biosciences). DNA concentration was quantified using a Qubit Fluorometer v4.0 (ThermoFisher Scientific) and the Qubit 1X dsDNA HS assay kit. Final library fragment size was assessed with the Agilent Femto Pulse Automated Pulsed Field CE Instrument (Agilent Technologies) using the gDNA 55 kb BAC analysis kit.

The sample was sequenced on a Revio instrument (Pacific Biosciences). The prepared library was normalised to 2 nM, and 15 μL was used for making complexes. Primers were annealed and polymerases bound to generate circularised complexes, following the manufacturer’s instructions. Complexes were purified using 1.2X SMRTbell beads, then diluted to the Revio loading concentration (200–300 pM) and spiked with a Revio sequencing internal control. The sample was sequenced on a Revio 25M SMRT cell. The SMRT Link software (Pacific Biosciences), a web-based workflow manager, was used to configure and monitor the run and to carry out primary and secondary data analysis.

### Hi-C

***Sample preparation and crosslinking***

The Hi-C sample was prepared from 20–50 mg of frozen whole organism tissue of the ilCelLacu2 sample using the Arima-HiC v2 kit (Arima Genomics). Following the manufacturer’s instructions, tissue was fixed and DNA crosslinked using TC buffer to a final formaldehyde concentration of 2%. The tissue was homogenised using the Diagnocine Power Masher-II. Crosslinked DNA was digested with a restriction enzyme master mix, biotinylated, and ligated. Clean-up was performed with SPRISelect beads before library preparation. DNA concentration was measured with the Qubit Fluorometer (Thermo Fisher Scientific) and Qubit HS Assay Kit. The biotinylation percentage was estimated using the Arima-HiC v2 QC beads.

***Hi-C library preparation and sequencing***

Biotinylated DNA constructs were fragmented using a Covaris E220 sonicator and size selected to 400–600 bp using SPRISelect beads. DNA was enriched with Arima-HiC v2 kit Enrichment beads. End repair, A-tailing, and adapter ligation were carried out with the NEBNext Ultra II DNA Library Prep Kit (New England Biolabs), following a modified protocol where library preparation occurs while DNA remains bound to the Enrichment beads. Library amplification was performed using KAPA HiFi HotStart mix and a custom Unique Dual Index (UDI) barcode set (Integrated DNA Technologies). Depending on sample concentration and biotinylation percentage determined at the crosslinking stage, libraries were amplified with 10–16 PCR cycles. Post-PCR clean-up was performed with SPRISelect beads. Libraries were quantified using the AccuClear Ultra High Sensitivity dsDNA Standards Assay Kit (Biotium) and a FLUOstar Omega plate reader (BMG Labtech).

Prior to sequencing, libraries were normalised to 10 ng/μL. Normalised libraries were quantified again and equimolar and/or weighted 2.8 nM pools. Pool concentrations were checked using the Agilent 4200 TapeStation (Agilent) with High Sensitivity D500 reagents before sequencing. Sequencing was performed using paired-end 150 bp reads on the Illumina NovaSeq 6000.

### Genome assembly

Prior to assembly of the PacBio HiFi reads, a database of *k*-mer counts ( *k* = 31) was generated from the filtered reads using FastK. GenomeScope2 ( Ranallo-Benavidez *et al.*, 2020) was used to analyse the *k*-mer frequency distributions, providing estimates of genome size, heterozygosity, and repeat content.

The HiFi reads were assembled using Hifiasm ( Cheng *et al.*, 2021) with the --primary option. The Hi-C reads ( Rao *et al.*, 2014) were mapped to the primary contigs using bwa-mem2 ( Vasimuddin *et al.*, 2019), and the contigs were scaffolded in YaHS ( Zhou *et al.*, 2023) with the --break option for handling potential misassemblies. The scaffolded assemblies were evaluated using Gfastats ( Formenti *et al.*, 2022), BUSCO ( Manni *et al.*, 2021) and MERQURY.FK ( Rhie *et al.*, 2020).

The mitochondrial genome was assembled using MitoHiFi ( Uliano-Silva *et al.*, 2023), which runs MitoFinder ( Allio *et al.*, 2020) and uses these annotations to select the final mitochondrial contig and to ensure the general quality of the sequence.

### Assembly curation

The assembly was decontaminated using the Assembly Screen for Cobionts and Contaminants ( ASCC) pipeline. TreeVal was used to generate the flat files and maps for use in curation. Manual curation was conducted primarily in PretextView and HiGlass ( Kerpedjiev *et al.*, 2018). Scaffolds were visually inspected and corrected as described by Howe *et al*. (2021). Manual corrections included 48 breaks and 53 joins. Chromosome Z was assigned based on synteny with the genome of *Hedya pruniana* (GCA_964197955.1). The curation process is documented at https://gitlab.com/wtsi-grit/rapid-curation. PretextSnapshot was used to generate a Hi-C contact map of the final assembly.

### Assembly quality assessment

The Merqury.FK tool ( Rhie *et al.*, 2020) was run in a Singularity container ( Kurtzer *et al.*, 2017) to evaluate *k*-mer completeness and assembly quality for the primary and alternate haplotypes using the *k*-mer databases ( *k* = 31) computed prior to genome assembly. The analysis outputs included assembly QV scores and completeness statistics.

The genome was analysed using the BlobToolKit pipeline, a Nextflow implementation of the earlier Snakemake version ( Challis *et al.*, 2020). The pipeline aligns PacBio reads using minimap2 ( Li, 2018) and SAMtools ( Danecek *et al.*, 2021) to generate coverage tracks. It runs BUSCO ( Manni *et al.*, 2021) using lineages identified by querying the GoaT database ( Challis *et al.*, 2023). For the three domain-level lineages, BUSCO genes are aligned to the UniProt Reference Proteomes database ( Bateman *et al.*, 2023) using DIAMOND blastp ( Buchfink *et al.*, 2021). The genome is divided into chunks based on the density of BUSCO genes from the closest taxonomic lineage, and each chunk is aligned to the UniProt Reference Proteomes database with DIAMOND blastx. Sequences without hits are chunked using seqtk and aligned to the NT database with blastn ( Altschul *et al.*, 1990). The BlobToolKit suite consolidates all outputs into a blobdir for visualisation. The BlobToolKit pipeline was developed using nf-core tooling ( Ewels *et al.*, 2020) and MultiQC ( Ewels *et al.*, 2016), with package management via Conda and Bioconda ( Grüning *et al.*, 2018), and containerisation through Docker ( Merkel, 2014) and Singularity ( Kurtzer *et al.*, 2017).

## Genome sequence report

### Sequence data

**Figure 2.**
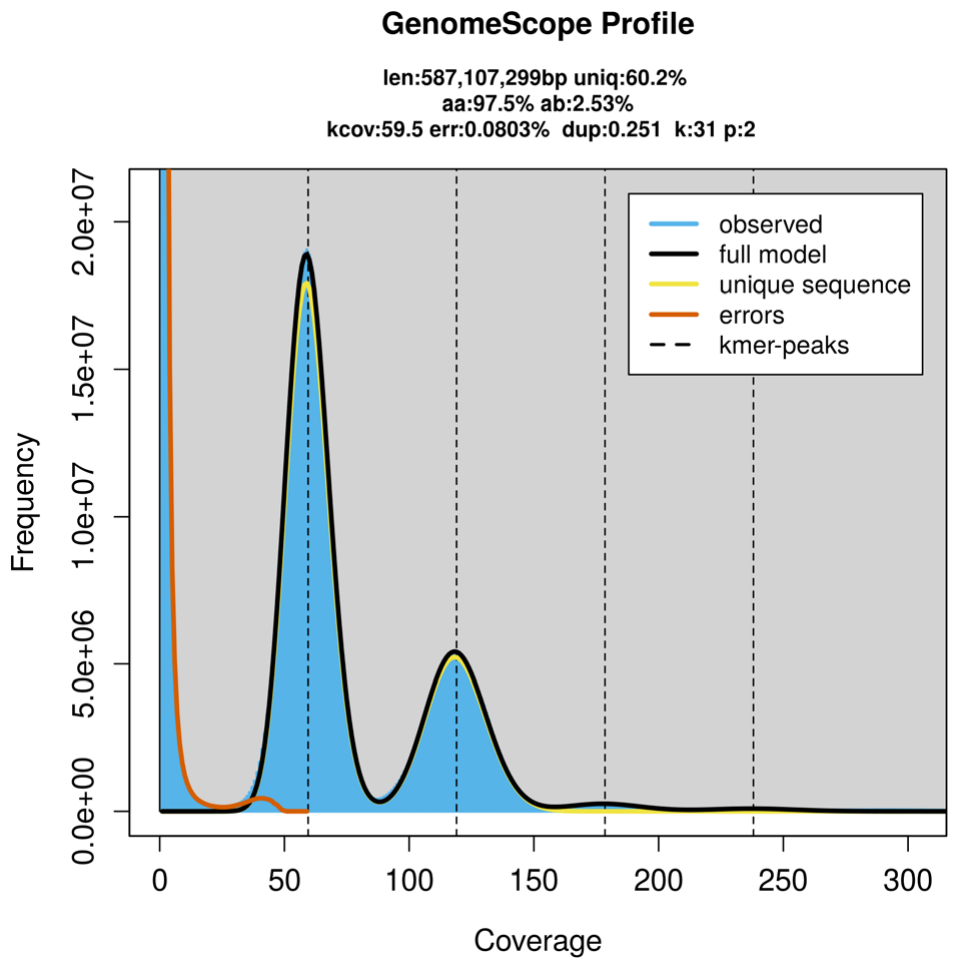
Frequency distribution of k-mers generated using GenomeScope2. The plot shows observed and modelled k-mer spectra, providing estimates of genome size, heterozygosity, and repeat content based on unassembled sequencing reads.

PacBio sequencing of the *Celypha lacunana* specimen generated 71.82 Gb (gigabases) from 6.38 million reads, which were used to assemble the genome. GenomeScope2.0 analysis estimated the haploid genome size at 587.11 Mb, with a heterozygosity of 2.53% and repeat content of 39.90% ( Figure 2). These estimates guided expectations for the assembly. Based on the estimated genome size, the sequencing data provided approximately 119× coverage. Hi-C sequencing produced 92.72 Gb from 614.05 million reads, which were used to scaffold the assembly. Table 1 summarises the specimen and sequencing details.

**Table 1.** Specimen and sequencing data for BioProject PRJEB76538.

Platform	PacBio HiFi	Hi-C
**ToLID**	ilCelLacu3	ilCelLacu2
**Specimen ID**	NHMUK015054506	NHMUK014536856
**BioSample (source individual)**	SAMEA114805555	SAMEA112221820
**BioSample (tissue)**	SAMEA114806839	SAMEA112221906
**Tissue**	whole organism	whole organism
**Sequencing platform and model**	Revio	Illumina NovaSeq 6000
**Run accessions**	ERR13265049	ERR13301087
**Read count total**	6.38 million	614.05 million
**Base count total**	71.82 Gb	92.72 Gb

### Assembly statistics

**Figure 3.**
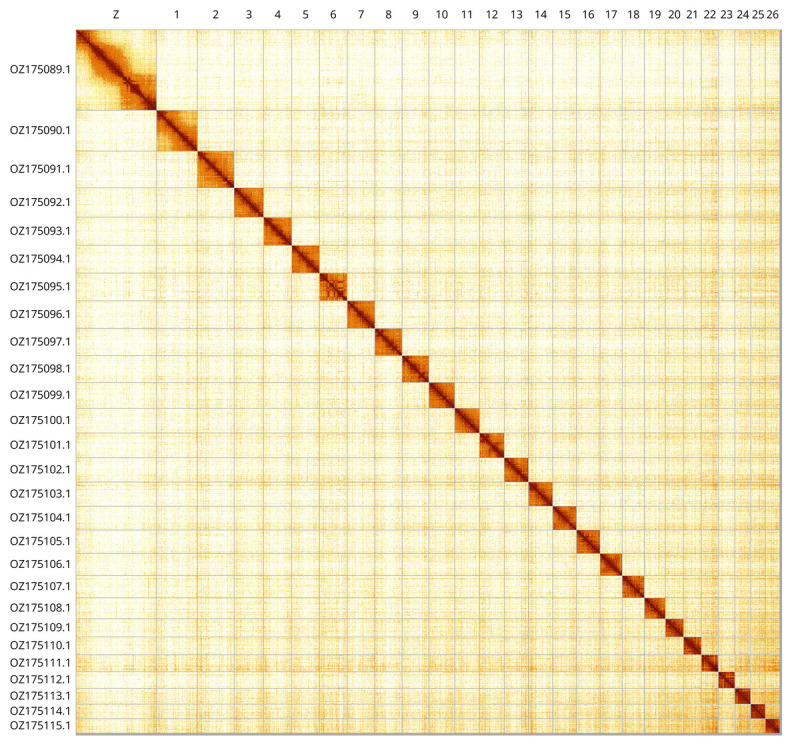
Hi-C contact map of the *Celypha lacunana* genome assembly. Assembled chromosomes are shown in order of size and labelled along the axes. The plot was generated using PretextSnapshot.

The primary haplotype was assembled, and contigs corresponding to an alternate haplotype were also deposited in INSDC databases. The final assembly has a total length of 591.83 Mb in 49 scaffolds, with 85 gaps, and a scaffold N50 of 21.54 Mb ( Table 2). Most of the assembly sequence (99.68%) was assigned to 27 chromosomal-level scaffolds. These chromosome-level scaffolds, confirmed by Hi-C data, are named according to size ( Figure 3; Table 3).

**Table 2.** Genome assembly statistics.

**Assembly name**	ilCelLacu3.1
**Assembly accession**	GCA_964237635.1
**Alternate haplotype accession**	GCA_964237625.1
**Assembly level**	chromosome
**Span (Mb)**	591.83
**Number of chromosomes**	27
**Number of contigs**	134
**Contig N50**	10.72 Mb
**Number of scaffolds**	49
**Scaffold N50**	21.54 Mb
**Sex chromosomes**	Z
**Organelles**	Mitochondrial genome: 17.21 kb

**Table 3.** Chromosomal pseudomolecules in the primary genome assembly of *Celypha lacunana* ilCelLacu3.

INSDC accession	Molecule	Length (Mb)	GC%
OZ175090.1	1	34.25	38
OZ175091.1	2	30.74	38
OZ175092.1	3	24.67	38.50
OZ175093.1	4	23.62	38.50
OZ175094.1	5	23.26	38.50
OZ175095.1	6	23.21	38.50
OZ175096.1	7	23.17	38.50
OZ175097.1	8	22.72	38.50
OZ175098.1	9	22.53	38
OZ175099.1	10	21.54	38.50
OZ175100.1	11	20.83	38
OZ175101.1	12	20.72	38.50
OZ175102.1	13	20.37	38.50
OZ175103.1	14	20.25	38.50
OZ175104.1	15	19.92	38.50
OZ175105.1	16	19.71	38
OZ175106.1	17	18.61	39
OZ175107.1	18	18.58	38.50
OZ175108.1	19	17.54	38.50
OZ175109.1	20	15.16	38.50
OZ175110.1	21	14.92	39
OZ175111.1	22	14.32	39.50
OZ175112.1	23	13.86	38.50
OZ175113.1	24	13.28	38.50
OZ175114.1	25	12.30	39
OZ175115.1	26	12.09	39.50
OZ175089.1	Z	67.74	38

The mitochondrial genome was also assembled. This sequence is included as a contig in the multifasta file of the genome submission and as a standalone record.

**Figure 4.**
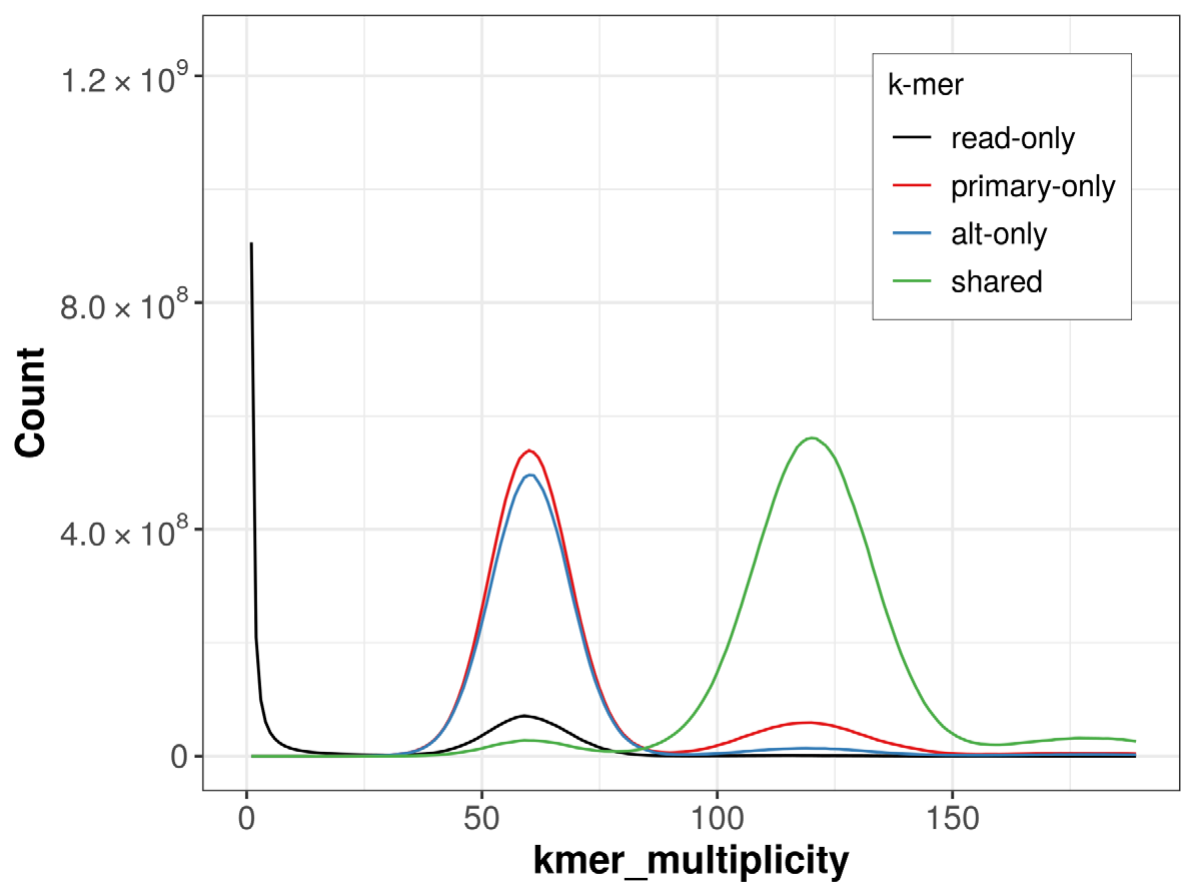
Evaluation of *k*-mer completeness using MerquryFK. This plot illustrates the recovery of *k*‐mers from the original read data in the final assemblies. The horizontal axis represents *k*‐mer multiplicity, and the vertical axis shows the number of *k*‐mers. The black curve represents *k*‐mers that appear in the reads but are not assembled. The green curve corresponds to *k*‐mers shared by both haplotypes, and the red and blue curves show *k*‐mers found only in one of the haplotypes.

The combined primary and alternate assemblies achieve an estimated QV of 64.7. The *k*-mer completeness is 65.35% for the primary assembly, 60.74% for the alternate haplotype, and 95.51% for the combined assemblies ( Figure 4).

**Figure 5.**
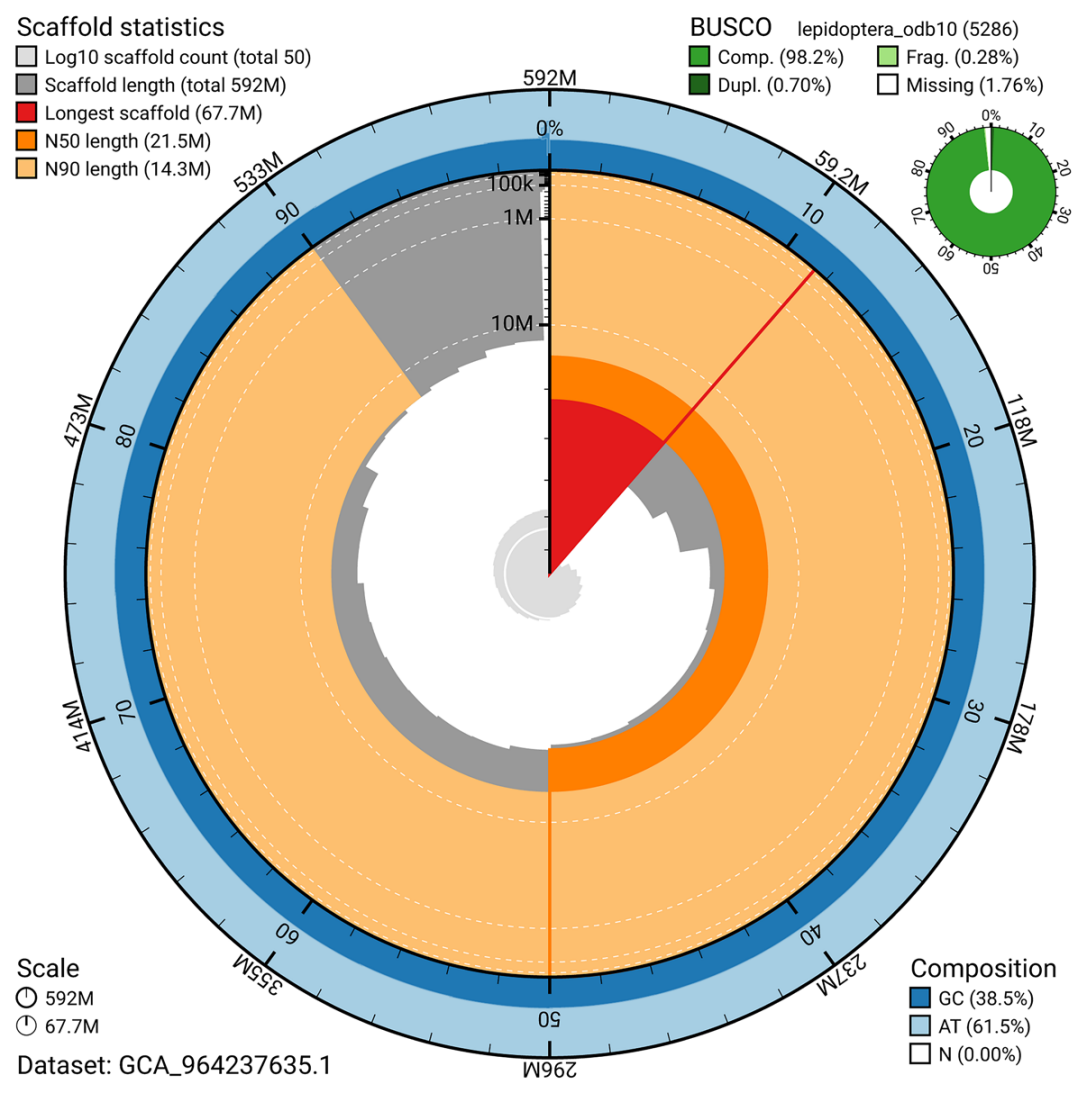
Assembly metrics for ilCelLacu3.1. The BlobToolKit snail plot provides an overview of assembly metrics and BUSCO gene completeness. The circumference represents the length of the whole genome sequence, and the main plot is divided into 1,000 bins around the circumference. The outermost blue tracks display the distribution of GC, AT, and N percentages across the bins. Scaffolds are arranged clockwise from longest to shortest and are depicted in dark grey. The longest scaffold is indicated by the red arc, and the deeper orange and pale orange arcs represent the N50 and N90 lengths. A light grey spiral at the centre shows the cumulative scaffold count on a logarithmic scale. A summary of complete, fragmented, duplicated, and missing BUSCO genes in the lepidoptera_odb10 set is presented at the top right. An interactive version of this figure can be accessed on the BlobToolKit viewer.

**Figure 6.**
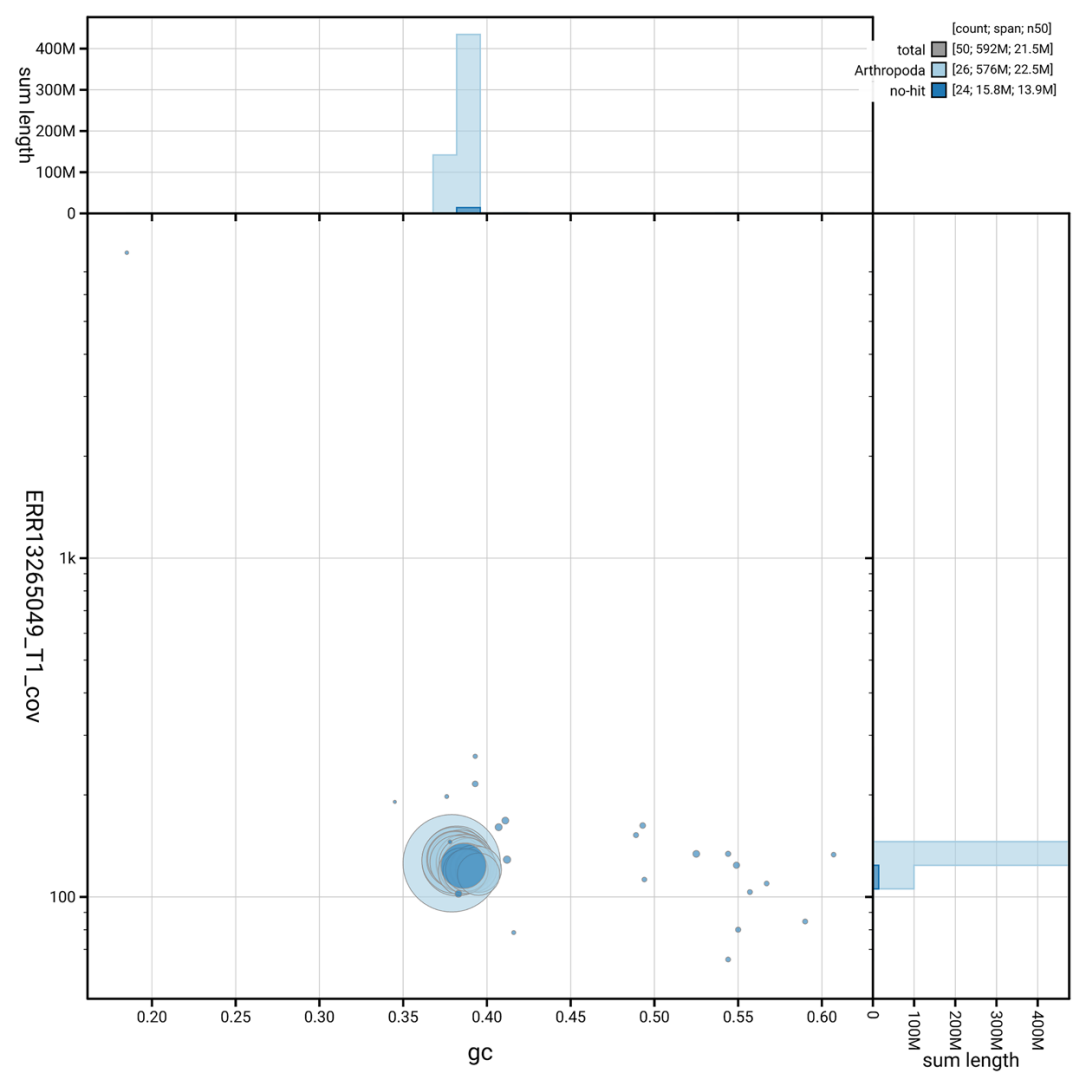
BlobToolKit GC-coverage plot for ilCelLacu3.1. Blob plot showing sequence coverage (vertical axis) and GC content (horizontal axis). The circles represent scaffolds, with the size proportional to scaffold length and the colour representing phylum membership. The histograms along the axes display the total length of sequences distributed across different levels of coverage and GC content. An interactive version of this figure is available on the BlobToolKit viewer.

BUSCO v.5.5.0 analysis using the lepidoptera_odb10 reference set ( *n* = 5 286) identified 98.2% of the expected gene set (single = 97.5%, duplicated = 0.7%). The snail plot in Figure 5 summarises the scaffold length distribution and other assembly statistics for the primary assembly. The blob plot in Figure 6 shows the distribution of scaffolds by GC proportion and coverage.

Table 4 lists the assembly metric benchmarks adapted from Rhie *et al.* (2021) the Earth BioGenome Project Report on Assembly Standards September 2024. The EBP metric, calculated for the primary assembly, is **7.C.Q66**, meeting the recommended reference standard.

**Table 4.** Earth Biogenome Project summary metrics for the *Celypha lacunana* assembly.

Measure	Value	Benchmark
EBP summary (primary)	7.C.Q66	6.C.Q40
Contig N50 length	10.72 Mb	≥ 1 Mb
Scaffold N50 length	21.54 Mb	= chromosome N50
Consensus quality (QV)	Primary: 66.0; alternate: 63.7; combined: 64.7	≥ 40
*k*-mer completeness	Primary: 65.35%; alternate: 60.74%; combined: 95.51%	≥ 95%
BUSCO	C:98.2% [S:97.5%; D:0.7%]; F:0.3%; M:1.5%; n:5 286	S > 90%; D < 5%
Percentage of assembly assigned to chromosomes	99.68%	≥ 90%

### Wellcome Sanger Institute – Legal and Governance

The materials that have contributed to this genome note have been supplied by a Darwin Tree of Life Partner. The submission of materials by a Darwin Tree of Life Partner is subject to the **‘Darwin Tree of Life Project Sampling Code of Practice’**, which can be found in full on the Darwin Tree of Life website. By agreeing with and signing up to the Sampling Code of Practice, the Darwin Tree of Life Partner agrees they will meet the legal and ethical requirements and standards set out within this document in respect of all samples acquired for, and supplied to, the Darwin Tree of Life Project. Further, the Wellcome Sanger Institute employs a process whereby due diligence is carried out proportionate to the nature of the materials themselves, and the circumstances under which they have been/are to be collected and provided for use. The purpose of this is to address and mitigate any potential legal and/or ethical implications of receipt and use of the materials as part of the research project, and to ensure that in doing so we align with best practice wherever possible. The overarching areas of consideration are:

- Ethical review of provenance and sourcing of the material
- Legality of collection, transfer and use (national and international)

Each transfer of samples is further undertaken according to a Research Collaboration Agreement or Material Transfer Agreement entered into by the Darwin Tree of Life Partner, Genome Research Limited (operating as the Wellcome Sanger Institute), and in some circumstances, other Darwin Tree of Life collaborators.

## Data Availability

European Nucleotide Archive: Celypha lacunana (common marble). Accession number PRJEB76538. The genome sequence is released openly for reuse. The *Celypha lacunana* genome sequencing initiative is part of the Darwin Tree of Life Project (PRJEB40665), the Sanger Institute Tree of Life Programme (PRJEB43745) and Project Psyche (PRJEB71705). All raw sequence data and the assembly have been deposited in INSDC databases. The genome will be annotated using available RNA-Seq data and presented through the Ensembl pipeline at the European Bioinformatics Institute. Raw data and assembly accession identifiers are reported in Table 1 and Table 2. Production code used in genome assembly at the WSI Tree of Life are available at https://github.com/sanger-tol. Table 5 lists software versions used in this study.

## Author information

Contributors are listed at the following links:

- Members of the Natural History Museum Genome Acquisition Lab
- Members of the Darwin Tree of Life Barcoding collective
- Members of the Wellcome Sanger Institute Tree of Life Management, Samples and Laboratory team
- Members of Wellcome Sanger Institute Scientific Operations – Sequencing Operations
- Members of the Wellcome Sanger Institute Tree of Life Core Informatics team
- Members of the Tree of Life Core Informatics collective
- Members of the Darwin Tree of Life Consortium

## References

[ref-1] Allio R Schomaker-Bastos A Romiguier J : MitoFinder: efficient automated large-scale extraction of mitogenomic data in target enrichment phylogenomics. *Mol Ecol Resour.* 2020;20(4):892–905. 10.1111/1755-0998.13160 32243090 PMC7497042

[ref-2] Altschul SF Gish W Miller W : Basic Local Alignment Search Tool. *J Mol Biol.* 1990;215(3):403–410. 10.1016/S0022-2836(05)80360-2 2231712

[ref-3] Bateman A Martin MJ Orchard S : UniProt: the Universal Protein Knowledgebase in 2023. *Nucleic Acids Res.* 2023;51(D1):D523–D531. 10.1093/nar/gkac1052 36408920 PMC9825514

[ref-4] British Lepidoptera: *Celypha lacunana*. 2025. Reference Source

[ref-5] Buchfink B Reuter K Drost HG : Sensitive protein alignments at Tree-of-Life scale using DIAMOND. *Nat Methods.* 2021;18(4):366–368. 10.1038/s41592-021-01101-x 33828273 PMC8026399

[ref-6] Bug Guide: Genus *Celypha*. 2025. Reference Source

[ref-7] Challis R Kumar S Sotero-Caio C : Genomes on a Tree (GoaT): a versatile, scalable search engine for genomic and sequencing project metadata across the eukaryotic Tree of Life [version 1; peer review: 2 approved]. *Wellcome Open Res.* 2023;8:24. 10.12688/wellcomeopenres.18658.1 36864925 PMC9971660

[ref-8] Challis R Richards E Rajan J : BlobToolKit – interactive quality assessment of genome assemblies. *G3 (Bethesda).* 2020;10(4):1361–1374. 10.1534/g3.119.400908 32071071 PMC7144090

[ref-9] Cheng H Concepcion GT Feng X : Haplotype-resolved *de novo* assembly using phased assembly graphs with hifiasm. *Nat Methods.* 2021;18(2):170–175. 10.1038/s41592-020-01056-5 33526886 PMC7961889

[ref-10] Crowley L Allen H Barnes I : A sampling strategy for genome sequencing the British terrestrial arthropod fauna [version 1; peer review: 2 approved]. *Wellcome Open Res.* 2023;8:123. 10.12688/wellcomeopenres.18925.1 37408610 PMC10318377

[ref-11] Danecek P Bonfield JK Liddle J : Twelve years of SAMtools and BCFtools. *GigaScience.* 2021;10(2): giab008. 10.1093/gigascience/giab008 33590861 PMC7931819

[ref-12] Ewels P Magnusson M Lundin S : MultiQC: summarize analysis results for multiple tools and samples in a single report. *Bioinformatics.* 2016;32(19):3047–3048. 10.1093/bioinformatics/btw354 27312411 PMC5039924

[ref-13] Ewels PA Peltzer A Fillinger S : The nf-core framework for community-curated bioinformatics pipelines. *Nat Biotechnol.* 2020;38(3):276–278. 10.1038/s41587-020-0439-x 32055031

[ref-14] Formenti G Abueg L Brajuka A : Gfastats: conversion, evaluation and manipulation of genome sequences using assembly graphs. *Bioinformatics.* 2022;38(17):4214–4216. 10.1093/bioinformatics/btac460 35799367 PMC9438950

[ref-15] GBIF Secretariat: *Celypha lacunana* (Denis & Schiffermüller, 1775). 2025. Reference Source 10.12688/wellcomeopenres.24736.1 PMC12481151 41036235

[ref-16] Gilligan TM Brown JW Baixeras J : Immigrant Tortricidae: Holarctic versus introduced species in North America. *Insects.* 2020;11(9):594. 10.3390/insects11090594 32899282 PMC7564570

[ref-17] Grüning B Dale R Sjödin A : Bioconda: sustainable and comprehensive software distribution for the life sciences. *Nat Methods.* 2018;15(7):475–476. 10.1038/s41592-018-0046-7 29967506 PMC11070151

[ref-18] Hancock EF : The moths and butterflies of Great Britain and Ireland, Volume 5, Part 2 Tortricidae: Olethreutinae.Leiden: E J Brill,2014. 10.1163/9789004264366_001

[ref-19] Howard C Denton A Jackson B : On the path to reference genomes for all biodiversity: lessons learned and laboratory protocols created in the Sanger Tree of Life core laboratory over the first 2000 species. *bioRxiv.* 2025. 10.1101/2025.04.11.648334 PMC12548527 41129326

[ref-20] Howe K Chow W Collins J : Significantly improving the quality of genome assemblies through curation. *GigaScience.* 2021;10(1): giaa153. 10.1093/gigascience/giaa153 33420778 PMC7794651

[ref-21] Kerpedjiev P Abdennur N Lekschas F : HiGlass: web-based visual exploration and analysis of genome interaction maps. *Genome Biol.* 2018;19(1): 125. 10.1186/s13059-018-1486-1 30143029 PMC6109259

[ref-22] Kurtzer GM Sochat V Bauer MW : Singularity: scientific containers for mobility of compute. *PLoS One.* 2017;12(5): e0177459. 10.1371/journal.pone.0177459 28494014 PMC5426675

[ref-23] Lawniczak MKN Davey RP Rajan J : Specimen and sample metadata standards for biodiversity genomics: a proposal from the Darwin Tree of Life project [version 1; peer review: 2 approved with reservations]. *Wellcome Open Res.* 2022;7:187. 10.12688/wellcomeopenres.17605.1

[ref-24] Li H : Minimap2: pairwise alignment for nucleotide sequences. *Bioinformatics.* 2018;34(18):3094–3100. 10.1093/bioinformatics/bty191 29750242 PMC6137996

[ref-25] Manni M Berkeley MR Seppey M : BUSCO update: novel and streamlined workflows along with broader and deeper phylogenetic coverage for scoring of eukaryotic, prokaryotic, and viral genomes. *Mol Biol Evol.* 2021;38(10):4647–4654. 10.1093/molbev/msab199 34320186 PMC8476166

[ref-26] Merkel D : Docker: lightweight Linux containers for consistent development and deployment. *Linux J.* 2014;2014(239): 2. Reference Source

[ref-27] Moth Dissection: Tortricidae: *Celypha lacunana*. 2025. Reference Source

[ref-28] Norfolk Moths: *Celypha lacunana* (Common Marble) - Norfolk Micro Moths - The micro moths of Norfolk. 2025. Reference Source

[ref-29] Ranallo-Benavidez TR Jaron KS Schatz MC : GenomeScope 2.0 and Smudgeplot for reference-free profiling of polyploid genomes. *Nat Commun.* 2020;11(1): 1432. 10.1038/s41467-020-14998-3 32188846 PMC7080791

[ref-30] Rao SSP Huntley MH Durand NC : A 3D map of the human genome at kilobase resolution reveals principles of chromatin looping. *Cell.* 2014;159(7):1665–1680. 10.1016/j.cell.2014.11.021 25497547 PMC5635824

[ref-31] Rhie A McCarthy SA Fedrigo O : Towards complete and error-free genome assemblies of all vertebrate species. *Nature.* 2021;592(7856):737–746. 10.1038/s41586-021-03451-0 33911273 PMC8081667

[ref-32] Rhie A Walenz BP Koren S : Merqury: reference-free quality, completeness, and phasing assessment for genome assemblies. *Genome Biol.* 2020;21(1): 245. 10.1186/s13059-020-02134-9 32928274 PMC7488777

[ref-33] Smart B : Micro-moth field tips: a guide to finding the early stages in Lancashire and Cheshire: Volume 2.Rishton: Lancashire & Cheshire Fauna Society,2021. Reference Source

[ref-34] Sterling P Parsons M Lewington R : Field guide to the micro-moths of Great Britain and Ireland: 2nd edition.London: Bloomsbury Wildlife Guides,2023. Reference Source

[ref-35] Twyford AD Beasley J Barnes I : A DNA barcoding framework for taxonomic verification in the Darwin Tree of Life project [version 1; peer review: 2 approved]. *Wellcome Open Res.* 2024;9:339. 10.12688/wellcomeopenres.21143.1 39386966 PMC11462125

[ref-36] UK Moths website: *Celypha lacunana* adult. 2025. Reference Source

[ref-37] Uliano-Silva M Ferreira JGRN Krasheninnikova K : MitoHiFi: a python pipeline for mitochondrial genome assembly from PacBio high fidelity reads. *BMC Bioinformatics.* 2023;24(1): 288. 10.1186/s12859-023-05385-y 37464285 PMC10354987

[ref-38] Vasimuddin M Misra S Li H : Efficient architecture-aware acceleration of BWA-MEM for multicore systems.In: *2019 IEEE International Parallel and Distributed Processing Symposium (IPDPS).*IEEE,2019;314–324. 10.1109/IPDPS.2019.00041

[ref-39] Wheeler JR : Micro moth vernacular names: a nomenclatural checklist of British Microlepidoptera.Norfolk: Clifton & Wheeler,2017. Reference Source

[ref-40] Zhou C McCarthy SA Durbin R : YaHS: yet another Hi-C Scaffolding tool. *Bioinformatics.* 2023;39(1): btac808. 10.1093/bioinformatics/btac808 36525368 PMC9848053

