# Supplementary material for: The genome sequence of the Common Marble, Celypha lacunana (Denis & Schiffermüller, 1775) (Lepidoptera: Tortricidae)
Source: Wellcome Open Res. 2025 Aug 11;10:428. [Version 1] doi: 10.12688/wellcomeopenres.24736.1 (PMC12481151; doi:10.12688/wellcomeopenres.24736.1)
Supplement: Peer review history

# Reviewer report: Reviewer response for version 1

**DOI:** 10.21956/wellcomeopenres.27253.r130861

**Published:** 2025-12-26

**Authors:** Henk den Bakker

**Affiliations:** 1 University of Georgia, Griffin, Griffin, USA

This manuscript describes the genome sequencing and background of a specimen of the Common Marble, a common tortrix moth in the British and Irish Isles. The  natural history (background) section is well written, as is the methods section. 

 One issue that should be addressed in my opinion is that the dataset is based on two specimens: ToLID ilCelLacu3 for the genome sequencing, and a second specimen, ToLID ilCelLacu2, for the Hi-C sequencing. It should be addressed why two specimens were needed (insufficient DNA yield from one specimen?). Was the identification verified by additional DNA barcoding for both specimens?

Are sufficient details of methods and materials provided to allow replication by others?

Partly

Is the rationale for creating the dataset(s) clearly described?

Yes

Are the datasets clearly presented in a useable and accessible format?

Yes

Are the protocols appropriate and is the work technically sound?

Yes

Reviewer Expertise:

Bioinformatics, systematics, genomics

I confirm that I have read this submission and believe that I have an appropriate level of expertise to confirm that it is of an acceptable scientific standard, however I have significant reservations, as outlined above.

# Reviewer report: Reviewer response for version 1

**DOI:** 10.21956/wellcomeopenres.27253.r131868

**Published:** 2025-09-29

**Authors:** Annabel Whibley

**Affiliations:** 1 The University of Auckland, Auckland, New Zealand; 2 Grapevine Improvement, Bragato Research Institute, Lincoln, New Zealand

In this Data Note, Gardiner and colleagues report the genome assembly of the **C**ommon Marble, *Celypha lacunana* a tortricid moth captured in a Natural England Natural History Museum BioBlitz for the  Darwin Tree of Life and UK Barcode of Life projects. Data inputs to the assembly follow the standard PacBio Hifi plus HiC sequence inputs. Whilst the Data Note follows the DToL template and has the expected clear and comprehensive structure and content, the natural history section of this report is particularly well-written. Public accession links are active.

 The assembly is of excellent quality: 7.C.Q66, with a total of 49 scaffolds. >99.5% of the assembly length was assigned to one of 27 chromosomes. This is no mean feat in a genome with >2.5% heterozygosity. While the gene completeness, as indicated by BUSCO, looks very good, the k-mer completeness falls a little short (~95% vs ~98%) suggesting that some of the more challenging regions of the genome may be marginally less well recovered.

Are sufficient details of methods and materials provided to allow replication by others?

Yes

Is the rationale for creating the dataset(s) clearly described?

Yes

Are the datasets clearly presented in a useable and accessible format?

Yes

Are the protocols appropriate and is the work technically sound?

Yes

Reviewer Expertise:

Genomics, Bioinformatics

I confirm that I have read this submission and believe that I have an appropriate level of expertise to confirm that it is of an acceptable scientific standard.

# Reviewer report: Reviewer response for version 1

**DOI:** 10.21956/wellcomeopenres.27253.r131870

**Published:** 2025-09-10

**Authors:** Kay Lucek

**Affiliations:** 1 University of Neuchâtel, Neuchâtel, Switzerland

The authors present the chromosome level genome assembly of a male specimen of the Common Marble moth *Celypha lacunana*. The assembly consists of 27 chromosomes and is not annotated for genes. The assembly is highly complete as revealed by the high BUSCO score but no information about phasing is given. Sequencing and genome assembly follow the current state of the art and use established methods. The assembly has been generated using the established pipelines of the Darwin Tree of Life Consortium.

 Overall, this is a nicely written genome note, especially for the biological and biogeographic background of the species. The presented assembly will be of great value for future biogeographic studies. An interesting fact to highlight is that Hi-C data was generated using an individual from another population. Some insights on how this may have affected the assembly would have been appreciated.

Are sufficient details of methods and materials provided to allow replication by others?

Yes

Is the rationale for creating the dataset(s) clearly described?

Yes

Are the datasets clearly presented in a useable and accessible format?

Yes

Are the protocols appropriate and is the work technically sound?

Yes

Reviewer Expertise:

Speciation genomics

I confirm that I have read this submission and believe that I have an appropriate level of expertise to confirm that it is of an acceptable scientific standard.
